# Detection of Virgin Olive Oil Adulteration Using Low Field Unilateral NMR

**DOI:** 10.3390/s140202028

**Published:** 2014-01-24

**Authors:** Zheng Xu, Robert H. Morris, Martin Bencsik, Michael I. Newton

**Affiliations:** 1 State Key Laboratory of Power Transmission Equipment & System Security and New Technology, Chongqing University, 174 Shazhengjie, Shapingba, Chongqing 400044, China; 2 School of Science and Technology, Nottingham Trent University, Clifton Lane, Nottingham, NG11 8NS, UK; E-Mails: rob.morris@ntu.ac.uk (R.H.M.); martin.bencsik@ntu.ac.uk (M.B.); michael.newton@ntu.ac.uk (M.I.N.)

**Keywords:** adulteration, olive oil, unilateral NMR Mouse, transverse relaxation, diffusion

## Abstract

The detection of adulteration in edible oils is a concern in the food industry, especially for the higher priced virgin olive oils. This article presents a low field unilateral nuclear magnetic resonance (NMR) method for the detection of the adulteration of virgin olive oil that can be performed through sealed bottles providing a non-destructive screening technique. Adulterations of an extra virgin olive oil with different percentages of sunflower oil and red palm oil were measured with a commercial unilateral instrument, the profile NMR-Mouse. The NMR signal was processed using a 2-dimensional Inverse Laplace transformation to analyze the transverse relaxation and self-diffusion behaviors of different oils. The obtained results demonstrated the feasibility of detecting adulterations of olive oil with percentages of at least 10% of sunflower and red palm oils.

## 1. Introduction

The detection of adulteration in high priced olive oils is a particular concern in the food industry. Virgin olive oil is simply pressed fruit without any additives and without the use of heat in the process; the best of these in terms of flavor are classified as extra virgin and must pass chemical tests in a laboratory and a sensory evaluation. The adulteration of extra virgin olive oil with other cheaper oils can lead to significant profits for the unscrupulous dealer. Laboratory-based methods have been extensively developed for the monitoring of adulteration of virgin olive oils with other edible oils [1–3]. Chromatographic methods [4] and mass spectrometry [5] have been used to study the adulteration of olive oil with hazelnut oil. Synchronous fluorescence spectroscopy was employed for quantitative determination of virgin olive oil adulteration with sunflower oil [6]. Priego Capote *et al.* developed an analytical method based on a gas chromatograph for the detection of extra virgin olive oil adulteration with four edible vegetable oils: sunflower, corn, peanut and coconut oils [7]. NMR spectroscopy was proven to be a much more effective method in the authentication of virgin olive oil based on their geographical origin [8]. For authentication purposes, several variables have been studied, including ^1^H, ^13^C-, and ^31^P-NMR analyses [9]. With more detailed NMR analyses, the diffusion coefficient of minor constituents of olive oil was chosen as a marker parameter [10,11]. All these methods have been employed with some success, but there are also some problems. Chromatographic methods of edible oil authentication have proved unable to detect adulterations at low concentrations [2]. Mass and NMR spectroscopy methods need large, expensive instrument, which are not suitable for screening. Fluorescence spectroscopy requires preprocessing operation to the samples and only Raman spectroscopy offers the possibility of a field portable instrument [12].

This article presents an alternative method, namely low field unilateral NMR, with the same potential as Raman for operation ‘in the field’. With this method, the transverse relaxation and self-diffusion of different adulterated oils have been studied. The samples can be measured without any preprocessing operation and the unilateral magnet structure means that the oils need not to be taken out from their original bottles, which can remain sealed.

## 2. Experimental Section

### 2.1. Samples

Sunflower oil (J. Sainsbury plc, Nottingham, UK) and red palm oil (Cebra Norfolk, Nottingham, UK) were used as the adulterations in the extra virgin olive oil (Napolina Ltd., Nottingham, UK). The samples were stored in the dark at room temperature until the day of analysis. Two mixtures of sunflower and extra virgin olive oils, with concentration of sunflower oil at two levels (10% and 20%), were prepared. The mixtures of sunflower and extra virgin olive oils were prepared using an Ultra-Turrax^®^ (IKA, Staufen, Germany). The red palm was gently heated in warm water prior to mixing by hand with the olive oil.

### 2.2. Instrument

**Figure 1.**
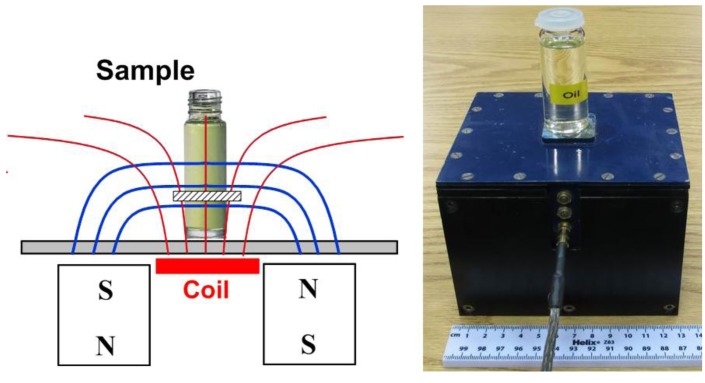
Schematic diagram of the NMR MOUSE with the sample and the selective slice shown.

NMR signals were acquired by using a NMR portable Mouse [13] from AixNMR (Wellington, New Zealand) with a Tecmag NMR spectrometer (Houston, TX, USA). The NMR Mouse takes advantage of a unilateral permanent magnet geometry that generates a magnetic field with a uniform static gradient at a defined distance from the magnet surface (Figure 1). In the sensitive volume, the static magnetic field has a strength of 0.25 T, and the gradient of 11.38 T/m. The thickness of the sensitive volume is 100 μm. The sensitive volume is made to reside 5 mm above the coil so will probe well inside a glass container of typical wall thickness.

### 2.3. Measurement and Data Processing

**Figure 2.**
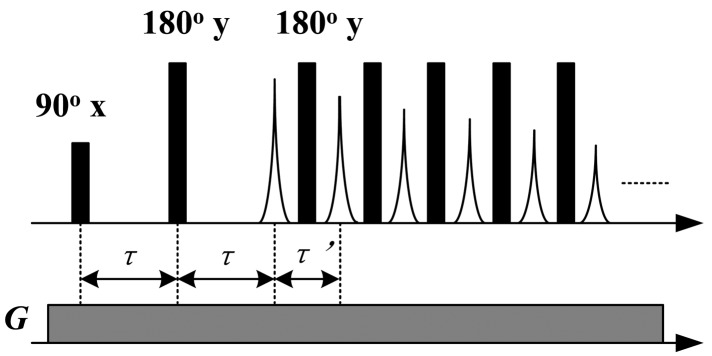
SGSE sequence composed of a Hahn echo and a CPMG sequence.

To detect the transverse relaxation and self-diffusion behaviors of oils, a SGSE [14] sequence as shown in Figure 2 was employed. The pulse widths of the 90° and the 180° pulses were 8 μs. The time interval between the 90° and the first 180° pulse (τ) was gradually increased from 120 μs to 2,500 μs for diffusion encoding. The echo time of CPMG sequence (τ') was fixed at 140 μs. The symbol *G* in Figure 2 corresponds to the constant gradient of the static magnetic field, which was 11.38 T/m and the RF frequency was 10.64 MHz. The CPMG echoes were recorded for further processing.

Due to the complex constituents of the samples studied, we expect to acquire multi-exponential signals for the transverse relaxation and diffusion. The recorded CPMG decay signals related to the two dimensional probability density distribution can be expressed as:

(1) $$M(\tau_{1},\tau_{2})\approx \iint k_{1}(T_{2},\tau_{1})k_{2}(D,\tau_{2})F(T_{2},D)dT_{2}dD+\varepsilon (\tau_{1},\tau_{2})$$

where, *k*_1_ is the kernel function of transverse relaxation which can be expressed as *e*^−^*^τ^*^1/^*^T^*^2^; *k*_2_ is the kernel function of diffusion which can be approximately expressed as $e^{-\frac{2}{3}\gamma^{2}G^{2}{\tau_{2}}^{3}D}$, γ is the gyromagnetic ratio of ^1^H, *G* is the constant gradient of the static magnetic field; *ε(τ*_1_, *τ*_2_*)* is a noise term. *F(T_2_*,*D)* is the two dimensional probability density distribution of *T*_2_ relaxation and diffusion. Although the CPMG echo train is attenuated by the diffusion, the small echo time of the CPMG sequence (τ' = 140 μs) alleviates this effect. We also neglected the Hahn echo attenuation which is related to T_2_. We developed in Matlab a 2-dimensional Laplace Inversion according to the algorithm of Venkataramanan *et al.* [15] to reconstruct *F(T_2_*,*D)* from the recorded CPMG decay signals.

## 3. Results and Discussion

### 3.1. Mixtures of Sunflower and Extra Virgin Olive Oils

**Figure 3.**
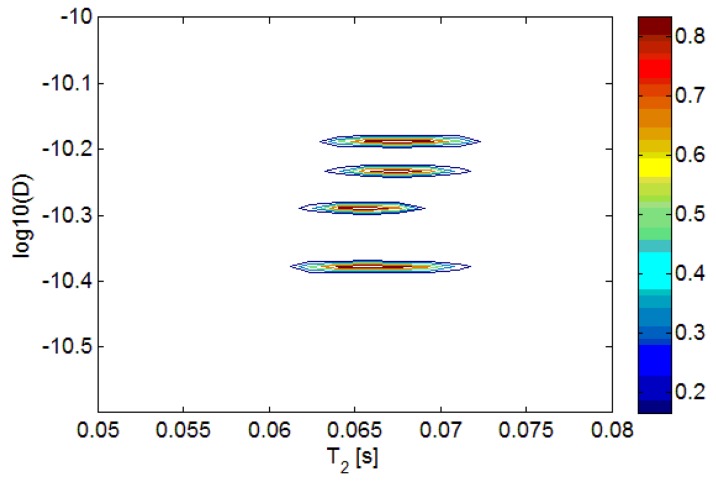
Superimposition of D-T_2_ distributions of sunflower and extra virgin olive mixed oils. From top to bottom, the peaks correspond to pure extra virgin olive oil, 10% sunflower oil, 20% sunflower oil and pure sunflower oil, respectively.

Two mixtures of sunflower and extra virgin olive oils, with sunflower concentrations at 10% and 20% by volume, pure sunflower oil and pure extra virgin olive oil, were measured. The D-T_2_ distributions of the different oils are plotted in Figure 3.

**Figure 4.**
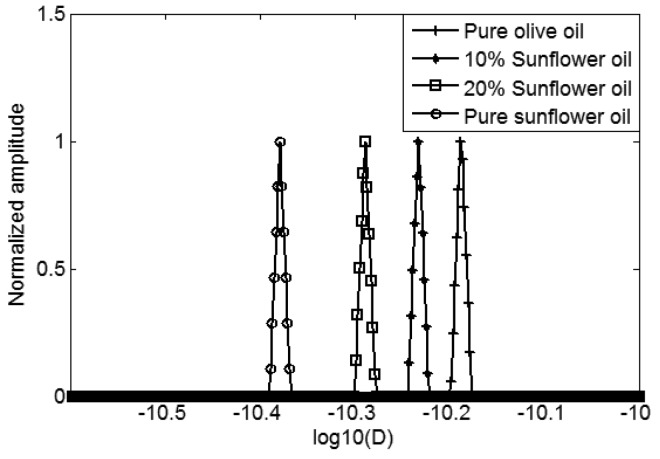
1D diffusion coefficient distribution of sunflower and extra virgin olive mixed oils.

In Figure 3, we can see that the D-T_2_ distribution plot of different oils separate in the vertical direction (D-axis), but overlap in the lateral direction (T_2_-axis). It indicates that the adulteration of sunflower oil in the extra virgin olive oil can more readily be detected from the sample's diffusion behavior. In order to better show the differences in diffusion, the 2-dimensional D-T_2_ distributions were projected to the D-axis, forming a 1-dimensional diffusion distribution, which is shown in Figure 4.

As shown in Figure 4, from right to left there are four peaks representing the pure extra virgin olive oil, 10% sunflower and 90% olive mixed oil, 20% sunflower and 80% olive mixed oil and the pure sunflower oil. The actual self-diffusion coefficients of these four oils are 6.5 × 10^−11^ m^2^s^−1^, 5.9 × 10^−11^ m^2^s^−1^, 5.1 × 10^−11^ m^2^s^−1^ and 4.1 × 10^−11^ m^2^s^−1^, respectively. Compared to the pure extra virgin olive oil, the sunflower oil mixtures have a smaller self-diffusion coefficient. The four narrow peaks in Figure 4 indicate that there are no diffusion distributions, and the diffusion has no correlation with the T_2_ relaxation. The food oil we have used are not similar to petroleum oils, for example, S3 [16], which has a D-T_2_ correlated distribution. The diffusion-relaxation behavior of oil is complex, although we do not see the diffusion distribution and its correlation with relaxation, it is still possible that, for other food oils, a diffusion distribution and correlation exist.

### 3.2. Mixtures of Red Palm and Extra Virgin Olive Oils

**Figure 5.**
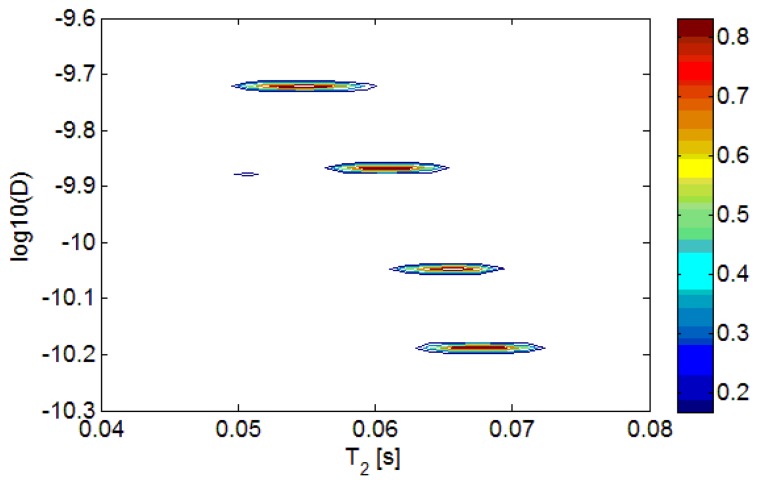
D-T_2_ distribution of red palm and extra virgin olive oil mixtures. From top to bottom, they are pure red palm oil, 10% red palm oil, 20% red palm oil and pure extra virgin olive oil, respectively.

**Figure 6.**
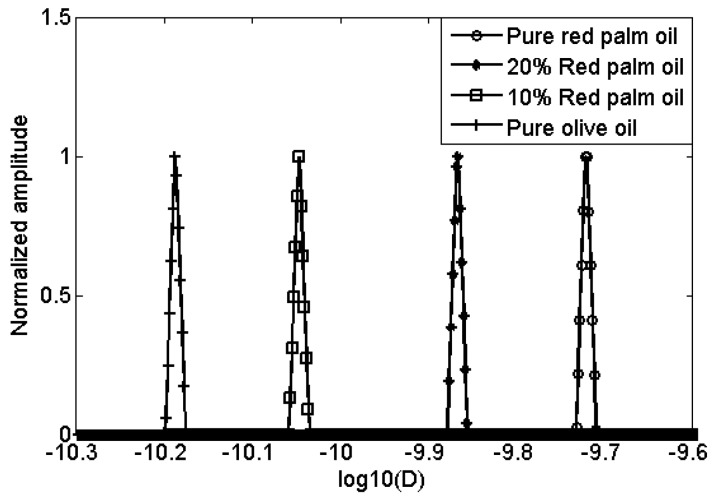
1d diffusion coefficient distribution of red palm and extra virgin olive mixed oils.

**Figure 7.**
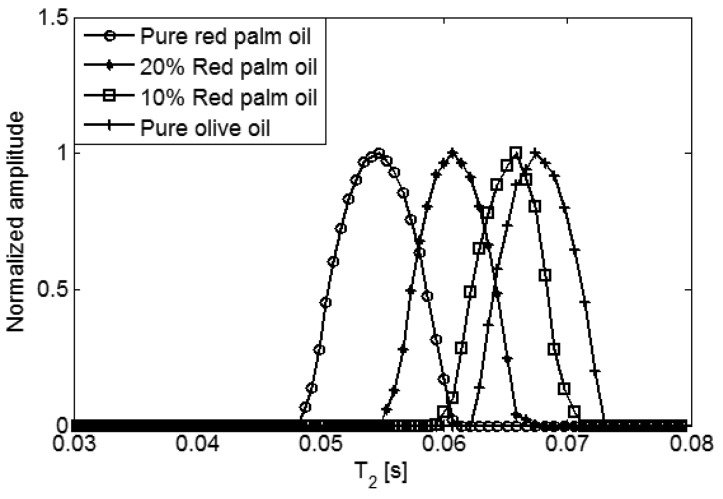
1d T_2_ distribution of red palm and extra virgin olive mixed oils.

Two mixtures of red palm and extra virgin olive oils, with concentration of red palm oil at two levels (10% and 20%), pure red palm oil and pure extra virgin olive oil, were also measured. The D-T_2_ distributions of these four different oils are plotted in Figure 5. For the red palm and extra virgin olive mixed oils, the distributions are separated not only in D-axis, but also in T_2_-axis. This indicates that the adulteration of red palm oil in the extra virgin olive oil can be detected both from the diffusion and transverse relaxation behaviors. To show the differences more clearly, the 2-dimensional D-T_2_ distributions were projected to the D-axis and T_2_-axis, as shown in Figures 6 and 7.

In Figure 6, from right to left there are four peaks representing the pure red palm oil, 10% red palm and 90% olive mixed oil, 20% red palm and 80% olive mixed oil and the pure extra virgin olive oil. The self-diffusion coefficient increases as the percentage of red palm oil increases, giving 6.5 × 10^−11^ m^2^s^−1^, 8.9 × 10^−11^ m^2^s^−1^, 1.4 × 10^−11^ m^2^s^−1^ and 1.9 × 10^−11^ m^2^s^−1^, respectively. While in Figure 7, the T_2_ decreases as the percentage of red palm oil increases, giving 67.5 ms, 64.1 ms, 60.8 ms and 54.9 ms, respectively. Comparing with the pure extra virgin olive oil, the red palm mixed oil has a larger self-diffusion coefficient but smaller T_2_.

## 4. Conclusions

This article demonstrates a low field unilateral NMR method to screen for the adulteration of extra virgin olive oil. With 2-dimensional Inverse Laplace transformation, the adulteration of extra virgin olive oil with different percentages of sunflower oil or red palm oil can be differentiated from the transverse relaxation and self-diffusion behaviors. The detection threshold is similar to that achieved using the much more expensive pulsed field gradient diffusion NMR measurement [10]. The narrow peaks in the diffusion distributions indicate that there is no distribution in the diffusion coefficients of our samples. It further suggests that the measurement of diffusion coefficient for the adulteration of olive oil can be sped up by just using two appropriate Hahn echo times (τ). Although the feasibility of this method has been demonstrated by these experiments, one problem was encountered. Measurements on the NMR MOUSE are temperature-sensitive because of the poor temperature coefficient of NdFeB magnets and an unstable static magnetic field influences the diffusion behavior measurement significantly. This however could be overcome using a temperature controlled environment or speeding up the measurement. Further work will concentrate of the ability of this technique to discriminate multiple additives.
